# Location-Based Lattice Mobility Model for Wireless Sensor Networks

**DOI:** 10.3390/s18124096

**Published:** 2018-11-22

**Authors:** Amer Al-Rahayfeh, Abdul Razaque, Yaser Jararweh, Muder Almiani

**Affiliations:** 1 College of Information Technology, Al-Hussein Bin Talal University, Ma’an, Jordan; amer.a.al-rahayfeh@ahu.edu.jo (A.A.-R.); malmiani@my.bridgeport.edu (M.A.); 2 Department of Computer Engineering & Telecommunication, International IT University, Almaty, Kazakhstan; arazaque@iitu.kz; 3 Jordan University of Science and Technology, Irbid 22110, Jordan

**Keywords:** wireless sensor networks, mobility, energy saving, disaster recovery, lattice mobility model, pattern

## Abstract

Significant research has been conducted for maintaining a high standard of communication and good coverage in wireless sensor networks (WSNs), but extra power consumption and mobility issues are not yet fully resolved. This paper introduces a memory-less location mobility-aware Lattice Mobility Model (LMM) for WSNs. LMM is capable of concurrently determining the node and sink mobility. LMM has a lower pause time, fewer control packets, and less node dependency (e.g., the energy consumed by each node in each cycle that is independent of the data traffic). LMM accurately determines a node’s moving location, the distance from its previous location to its current location, and the distance from its existing location to its destination. Many existing mobility models only provide a model how nodes move (e.g., to mimic pedestrian behavior), but do not actually control the next position based on properties of the underlying network topology. To determine the strength of LMM, OMNet++ was used to generate the realistic scenario to safeguard the affected area. The operation in affected area comprises searching for, detecting, and saving survivors. Currently, this process involves a time-consuming, manual search of the disaster area. This contribution aims to identify an energy efficient mobility model for a walking pattern in this particular scenario. LMM outperforms other mobility models, including the geographic-based circular mobility model (CMM), the random waypoint mobility model (RWMM) and the wind mobility model (WMM), The simulation results also demonstrate that the LMM requires the least time to change the location, has a lower drop rate, and has more residual energy savings than do the WMM, RWMM, and CMM.

## 1. Introduction

The wireless sensor networks experience problems for applications that require mobility support, such as disaster/earthquake recovery, battlefield surveillance, monitoring and handling animals, and law enforcement situations. These applications require a dynamically adoptable WSN network with mobility support to guarantee reliable communication, reliable network connectivity, low energy consumption and a longer network lifetime [1].

Thus, it is essential to introduce a system that is energy efficient at all levels of the protocol stack. The energy efficiency and scalability of WSNs depend on the mobility variances [2]. As a result, the lack of a robust mobility model results in performance degradation and unpredictable broken links [3].

Furthermore, mobility plays a significant role in identifying the time-based features of network traffic in WSNs that directly affect resource provision structures and enable network designed control. It has been confirmed that high traffic load posed by a single sensor node is narrowly associated with the mobility inconsistency of that node and the spatial connection with the observed phenomenon [4].

Bursty traffic is more likely in a scenario with high mobility variations and low spatial links. Conversely, balanced traffic is more likely when mobility discrepancy decreases and spatial correlation upsurges. These observations point out that certain mobility characteristics can produce the desired outcome in different temporal constructions of network traffic. On the other hand, mobility-based WSNs are usually preferable over static WSNs because they result in better energy efficiency, enhanced coverage, improved object tracking, and higher channel capacity [5]. There are several WSN applications where nodes should be capable of adopting mobility, which require proper consideration [6]. The lack of mobility support also highly distracts the routing performance in WSNs. As a result, network topology changes unpredictably and paths frequently fail, which may increase packet delay and packet loss. Moreover, the QoS provisioning at the lower layers and the transport layer relatively correlates with the presence of mobility. Otherwise, sensor nodes experience the issues of emitting, increased latency, jitter and congestion.

There are several mobility models available in the literature, all of which are particularly good candidates for ad-hoc WSNs, but their behavior in WSNs worsens QoS parameters and results in high energy consumption. For example, RWMM performs reasonably well in ad-hoc wireless networks, but is a poor candidate for WSNs due to weak choice of velocity distribution and uniform distribution [7,8,9]. To overcome the shortcomings of RWMM, the nomadic community mobility model (NCM) was introduced. This model is suitable for military applications and mobile communication in conferences because nodes move randomly as a group from one location to another. However, the reference point of each node is identified based on the overall movement of the group [10,11]. This model consumes an excess energy when determining the location of a single node.

To handle the mobility of sink nodes, CMM was introduced. This model captures data while following a circular pathway that is based on static points. A major shortcoming of this model is that nodes must remain motionless for eight or sixteen times in each cycle. In order to extend the network lifetime, WMM, based on eight directions, was introduced [12], but this model is specifically designed for sink nodes, but nodes have only group mobility support. Consequently, in determining the location of a single node, group nodes move and consume energy [13]. As a result, WMM mobility model suffers due to the extra pause time, the speed of nodes, and the dependency of nodes- movement in the group causes the network performance to slow down [14]. Furthermore, existing mobility models are fit for determining the sink mobility, but experience problems in the case of node mobility. The state-of-art in this research is to maintain QoS provisioning and prolong the network lifetime. To improve the performance of WSNs, this paper introduces the LMM. The LMM has features of handling the upward, lateral, and even downward movement. For example, a sensor node may be promoted to be a sink node, but to continue to work as a node at the same time, the node may need to take a lateral step to gain knowledge regarding other nodes for sending and receiving the data (e.g., schedules, priorities). After gaining these details, the node may then become a sink node again, this time in measuring the energy rather than distance and location. The LMM handles the limitations of existing models and makes the following contributions:

- Handling the node and sink mobility concurrently.
- Reducing control packets when detecting the location of nodes.
- Decreasing extra pause time.
- Determining the node’s distance from the previous to the current location, on variance mobility rates.

The remaining sections are organized as follows: Section 2 gives an overview on the state of the art research. Section 3 highlights the existing problem and mobility model formulation. Section 4 describes the simulation setup and the analysis of results, Section 5 gives the discussion of the results and finally Section 6 concludes the work.

## 2. State of the Art Overview

There are several WSN applications such as disaster/earthquake recovery, battlefield surveillance, and monitoring bird and animal activities, which require the availability of strong mobility features. Understanding the mobility models in a realistic environment is a challenging task. In the real world, mobility is of paramount significance. Considering the behavior of the real-world entities, mobility models are categorized as synthetic or trace-based [15]. Synthetic models scale the real-world mobile objects. However, these models are unable to show an exact behavior of movable patterns. On the other hand, trace-based models involve a large number of contestants for a longer monitoring period, but the real trajectory movement of mobile nodes is hard to capture. The above is concerning due to the deployment of emerging applications for mobility-aware environments. In particular, when sensor nodes are moving, it is challenging to maintain the QoS provisioning and prolong the network lifetime.

Many researchers have focused on deploying the mobile nodes in WSNs to extend the network lifetime and improve the performance. As a result, there are some of the interesting contributions focusing on the mobility available in the literature. However, majority of the existing contributions in mobility models use the single mobile node to gather the information from stationary nodes. Most of the existing multi-node supported mobility models focus on the mobile sensor nodes but lack the mobile-sink support. One of interesting contributions focusing on the Modified De Bruijn graph (MDBG) model is introduced to support mobile sinks [16]. The model helps collect the data from static sensor nodes. This model aims to combine single and multi-hop communication for decreasing the end-to-end delay, but did not focus on the accuracy. Handling this issue, the extended Mobility Markov Chain model is introduced [17] to combine the n preceding visited locations in order to improve the accuracy when determining the next predictable location. However, the model did not control the movement of individual sensor nodes. The authors in [18] introduced a two-tier autoregressive group mobility model to handle the individual sensor nodes’ movement by using a first-tier. Furthermore, the group mobility behavior is apprehended in the second tier by using the mobility correlation of nodes. As a result, the group is acknowledged by applying the correlation index test. In addition, the model estimates the group-based mobility where representative mobility-aware node is used to calculate the mobility state of the group members. On the other hand, the two-tier autoregressive group mobility model is unable to accumulate the complete information of the clusters. In [19], a mobility model for self-configuring mobile sensor networks is introduced to calculate the mobility patterns of mobile sensor nodes applying artificial potential functions and cluster formation. The mobility is calculated by applying two components: particle and potential field, which are based on artificial potential functions. This model lacks support for determining the mobility of individual nodes. Thus, the topology-based mobility model is proposed to support main aspects of the random waypoint mobility model and random-way-walk mobility model [20]. The mobility model is incorporated with a lightweight medium access control protocol to achieve the objects of random walking patterns. This model handles each mobile sensor node individually, but increases the number of the control messages. The simple truncated Levy walk mobility model is introduced to capture the tendency of the people that move within predefined area of routine activities [21]. The simple truncated Levy walk mobility model reduced the control messages by using emulation. However, the model is used for mobile ad hoc networks and delay-tolerant networks. In [22], static sink and mobile sink are comprehensively described that collect the data by ending the locality of the sensor nodes. In the first step, performance of three different approaches is investigated in order to design and develop sink mobility models for energy efficient data gathering and delay-tolerant static wireless sensor networks. In the second step, a sensor node is randomly chosen based on the maximum number of exposed neighbor nodes. Finally, the authors recommend to use random node selection-based strategy for sink mobility models instead of keeping the track of the number of exposed neighbor nodes per node and available residual energy of the sensor node. These existing mobility models either focus on maintaining the connectivity or throughput performance. Whereas, our proposed lattice location-based mobility model concurrently determines the node and sink mobility for improving the energy efficiency and QoS provisioning. It reduces the pause time, number of control packets and node dependency. Furthermore, it also detects the node’s moving location, the distance from its previous location to its current location, and the distance from its existing location to its destination.

## 3. Problem Statement and Lattice Mobility Model Formulation

The limitations postured by the mobility of WSNs greatly interrupt the smooth communication process. Moreover, it is quite difficult to concurrently address the problems of node and sink mobility. The key question is how to enable WSNs to efficiently maintain QoS provisioning and extend the network lifetime with respect to imposed mobility limitations for robust disaster/earthquake salvage operations.

In a WSN, the mobility behavior in the mobile sensor node can be categorized by a two-phase process—an active and an inactive phase. During an active phase, the compressed data are transmitted to other nodes or sinks. During an inactive phase, a mobile sensor node moves to a new location, then collects and locally compresses data. To frame this mobility behavior, we introduce the LMM to control the random movement of sensor nodes over WSNs. The idea of the lattice comes from a moderately ordered set in which every pair of elements is based on a supremum (known as a join or least upper bound) and an infimum (known as a meet or greatest lower bound). For example, the network ‘*n*’ consists of different regions and supremum can be considered as a subset ‘$S_{s}$’ or a network region-1 ‘*n*’, but it could be partially ordered set of another region ‘*R*’. Thus, nodes in ‘$S_{s}$’ are referred to as least element in R. The nodes in another region ‘*R*’ are greater than or equal to all the existing elements of ‘$S_{s}$’. As a result, supremum is also referred to as the least upper bound (LUB). 

**Figure 1:**
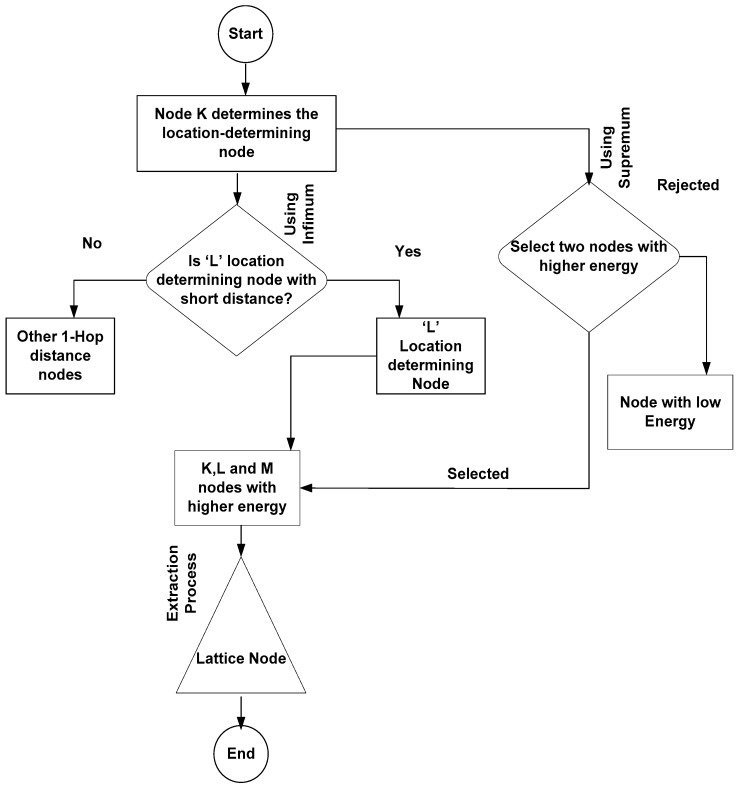
Lattice node selection process based on infimum and supremum properties.

On other hand, infimum is the subset ‘$S_{s1}$’ or a network region-2 that is partially ordered set of another region ‘*R*’, but the nodes of ‘$S_{s}$’ are greatest elements in ‘R’ that are less than or equal to all elements of ‘$S_{s1}$’. As a result, infimum is referred to as greatest lower bound (GLB). Thus, an idea of supremum and infimum is applied in the LMM, which helps the sensor nodes maintain connectivity and choose the node with more energy and the shortest distance during the communication. We choose the specific node for targeting the mobility-aware node based on the least distance and high residual energy using the concept of infimum and supremum, as depicted in Figure 1. We formulate the problem using supremum and infimum. The supremum is used to represent and sample the maximum energy of senor node in the region ‘*R*’ and infimum is used for determining the shortest distance of node available at 1-hop neighborhood. Let ‘*K*’ be a sensor node that wants to find the location and distance of communicating node. Thus, node ‘*K*’ uses infimum features to determine the 1-hop neighbor distance node that is called as the location-determining node ‘*L*’. ‘*L*’ is the closest node to ‘*K*’ in the region ‘*R*’ in the network ‘*N*’. In addition, we initially pick three nodes, including ‘*K*’ in the same region with maximum energy. These nodes are ‘*K*’, ‘*L*’ and ‘*M*’ with the highest residual energy. The nodes are picked using supremum features for determining the highest energy available in the node at 1-hop neighbor distance. Based on infimum and supremum, the next node is selected to be responsible for forwarding the data and finding the location of desired destination node explained in Figure 1.

**Figure 2:**
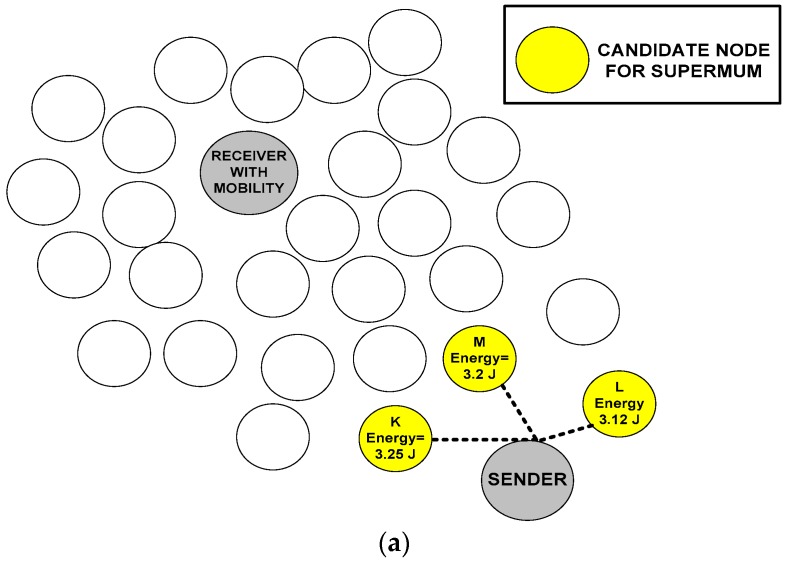

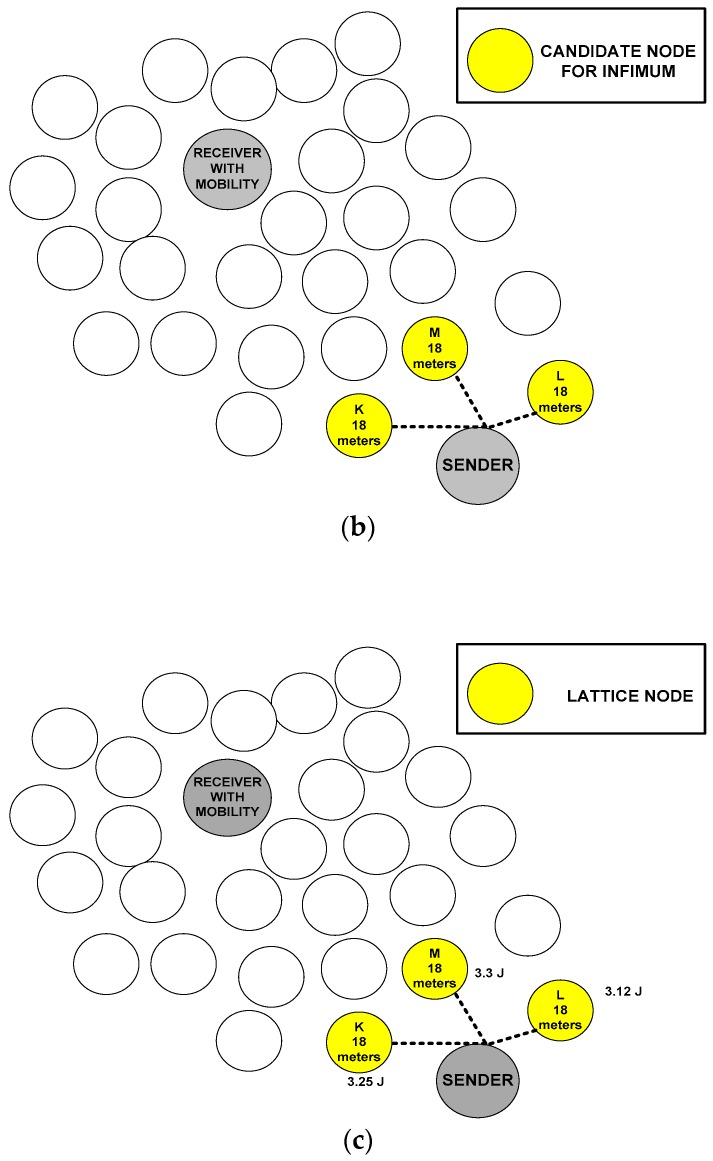
(**a**) Selection of the supremum node based on the highest residual energy using the upper bound. (**b**) Selection of the infimum node based on the shortest distance using the greatest lower bound. (**c**) Selection of the lattice node based on the shortest distance and highest residual energy using greatest lower bound and upper bound, respectively (satisfying both conditions of supremum and infimum).

The chosen node is called a lattice node. Figure 2 gives a graphical representation of determining the lattice node.

In Figure 2b, the final lattice node is the one that satisfies both conditions of supremum and infimum. If the sensor node ‘*K*’ is already proved with maximum energy (supremum), then this claim can furthermore be satisfied using Theorem 1.

**Theorem** **1.** *Every nonempty search of ‘K’ node with below infimum and above supremum in the region ‘R’ is bounded.*

**Proof.**  This theorem provides the foundation for many existence results in realistic scenarios and studies. For example, once we validate that a sensor node is bounded from above, then we may prove the existence of the supremum without determining its value.

The infimum and supremum sensor node is the lattice node in the region ‘*R*’, which is unique, if it exists. Additionally, if both the supremum and infimum exist, then Rinfmum ≤ RSupmum. □

**Proof.**  Assume that a sensor node ‘*K*’ is the supremum in the region ‘R’. Then *K* ≥ *γ*; thus, a sensor node ‘*K*’ is the upper bound in the region ‘*R*’. Similarly, if ‘*K*’ is the infimum sensor node in the region ‘R’, then *K* ≤ *γ*. Thus, ‘*K*’ is the greatest lower bound in the region ‘*R*’. It can be expressed as follows:

If *K* ≥ *γ*, then *K* = *γ*.

If Rinfmum and RSupmum exist, then the region ‘*R*’ is nonempty. Thus, select ‘*K*’ sensor node that is the nearest node with the highest residual energy. It can be written as:

*K* ∈ *R*, &Rinfmum ≤ *K* ≤ RSupmum.

Rinfmum and RSupmum are the greatest lower bound and upper bound respectively in the region ‘*R*’. It follows that Rinfmum ≤ RSupmum.

If RSupmum ∈ *R*, then it is represented as Rhigh and called the highest residual energy of the node ‘*K*’ in region ‘*R*.’ If Rinfmum ∈ *R*, then it is represented as Rlow and called the shortest distance of node ‘*K*’ from location-determining node ‘*L*’ in the region ‘*R*’. □

Based on the concept of infimum and supremum, LMM searches for the unique node with the highest residual energy and the shortest distance from the source node to the destination node.

The idea of infimum and supremum is applied with LMM to support three types of mobility patterns: dynamic medium mobility patterns, walking mobility patterns, and vehicular mobility patterns. Dynamic medium mobility patterns are used when the sensor nodes are in a medium (e.g., wind, water, or other fluid) and typically supports two or three dimensions. Walking mobility patterns are suitable for people; movements in these patterns are two dimensional. These patterns are characterized by restricted speeds, a chaotic nature, and obstacle avoidance. Vehicular mobility patterns support vehicles that are equipped with sensor nodes. The vehicles communicate by exchanging traffic conditions with each other. The movement of the vehicle is measured in only one direction.

**Theorem** **2.** *The greatest lower bound is constructed on the infimum. Let V = (a, t) be the speed of the mobile sensor node at the given time ‘t’ and at the location $X=(X_{1},\;X_{2}).$*

**Proof.**  In the greatest lower bound, *D* = (*a*, *t*) is the node distribution along the direction ‘∆*di*’. Each sensor node has three nearest neighbor nodes as:

(1) $$a_{i}+R.\;∆di(\;\gamma =1,\;2,\;3)$$

where ‘*R*’ is the region of the network. Thus, different direction ‘∆*di*’ of neighbor node ‘*a*’ in the region ‘*R*’ can be defined as follows:

(2) ∆di={1i=1π2cos(i),π2sin(i)i=2,3

Greatest lower bound (Infimum) can be governed by following equation:

(3) $$D_{i}\left(a_{i}+R.\;∆di,\;t+R\right)-D_{i}\left(a,t\right)=\tau_{i}\left(V\right).\left[D_{I}{}^{\beta }\left(a,t\right)-D_{i}\left(a,t\right)\right]\left(i=1,2,3\right)$$

where ‘$D_{I}{}^{\beta }$’ is the distribution of the nodes that can be predicted by the value of $D_{i}\left(a,t\right)$; we set $D_{I}{}^{\beta }\left(a,t\right)$ to be an average speed of v=(a,t):

(4) $$D_{I}{}^{\beta }\left(a,t\right)=\frac{V\left(a,t\right)}{3}\left(i=1,2,3\right)$$

where ‘*i*’ indicates the number of the mobile sensor nodes having an average speed.

The greatest lower bound requires that speed of mobile sensor node is measured during the progression. Mathematically, it entails that the summation of the left-hand side of Equation (3) over all three neighborhoods must be equal to 1:

(5) $$\overset{3}{\underset{i=1}{\sum }}\tau_{i}\left(V\right).[D_{I}{}^{\beta }\left(a,t\right)-D_{i}\left(a,t\right)]=1$$

Substituting Equation (5) into Equation (4), we get:

(6) $$V=\left(a,t\right)=\overset{3}{\underset{i=1}{\sum }}\tau_{i}\left(V\right).\;D_{i}\left(a,t\right)\overset{3}{\underset{i=1}{\sum }}\frac{\tau_{i}\left(V\right)}{3}=\overset{3}{\underset{i=1}{\sum }}D_{I}{}^{\beta }\left(a,t\right)$$

 □

Thus, we summarize the result of the theorem based on the greatest lower bound (infimum) in the algorithm given as:

**Algorithm 1:** The greatest lower bound (Infimum).(a). For all neighbor nodes $D_{i}\left(a,\;K+1\right)=D_{i}\left(a,\;K\right)+\tau_{i}\left(V\right).\left[\frac{V\left(a,\;K\right)}{3}-D_{i}\left(a,\;K\right)\right]\left(i=1,2,3\right)$ (b). For each neighbor node $D_{i}\left(a+∆di,\;K+1\right)=D_{i}\left(a,\;K+1\right)\;\left(i=1,2,3\right)$ (c). For each border node $D_{i}\left(a+∆di,\;K+1\right)=D_{i}\left(a+∆d_{i+1},K+1\right)\left(i=1,2,3\right)$ Here, boarder node functions as head node to collect the data from other nodes that are within reach. (d). Update the speed for all moving nodes $V=\left(a,K+1\right)=\overset{3}{\underset{i=1}{\sum }}\tau_{i}\left(V\right).\;D_{i}\left(a,K+1\right)/\overset{3}{\underset{i=1}{\sum }}\frac{\tau_{i}\left(V\right)}{3}$

The goal of this contribution is to focus on the walking mobility patterns because no existing mobility model supports walking patterns well. Hence, RWMM and the group mobility model have few capable features that can be modified to support many scenarios [23]. However, both are closer to ad-hoc and wireless networks than WSNs. LMM helps increase throughput, increase energy efficiency and reduce latency. Let us define how to use LMM in region based WSNs to handle walking mobility patterns:

Let ‘*K*’ be a sensor node that reaches location ‘*L*’. The probability of determining the location ‘*PK* (*L*)’ of the sensor node can be defined as:

(7) $$L=K_{1}x_{1}+K_{2}x_{2}+K_{3}x_{3}+K_{4}x_{4}+,\ldots ,K_{n}$$

Equation (7) shows the number of hops from originating node to destination node for determining the location of the desired node.

Assume that the sensor nodes in the lattice are periodic with a periodic location ‘*L*’. Then no boundary condition exists, so the probability ‘$P_{K}\left(L\right)$’ of determining the location of the sensor node can be calculated using the following recurrence formula:

(8) $$P_{K+1}\left(L\right)=\underset{Ĺ}{\sum }T\left(L-Ĺ\right)P_{K}\left(Ĺ\right)$$

where T(L−Ĺ) is the probability of the sensor node when it moves from location ‘*L*’ to location ‘Ĺ’. We introduce a generating function for the moving sensor node as follows:

(9) $$\psi \left(L,\;T_{n}\right)=\overset{\infty }{\underset{K=0}{\sum }}T_{n}{}^{K}P_{K}\left(L\right)$$

where ‘$T_{n}$’ is the trajectory of the sensor node and ‘ψ’ is the state of the node in WSNs.

We express the case in which the sensor node starts moving from the origin to another destination as

(10) $$\left(L,\;T_{n}\right)=-T_{n}\underset{Ĺ}{\sum }T\left(L-Ĺ\right)\psi \left(Ĺ,\;T_{n}\right)=\delta_{L,0}$$

(11) $$P_{0}\left(L\right)=\gamma_{L,0}=\{\begin{array}{c} 1,\;L=0,\\ 0,\;otherwise. \end{array}$$

where δ is the Kronecker delta.

**Figure 3:**
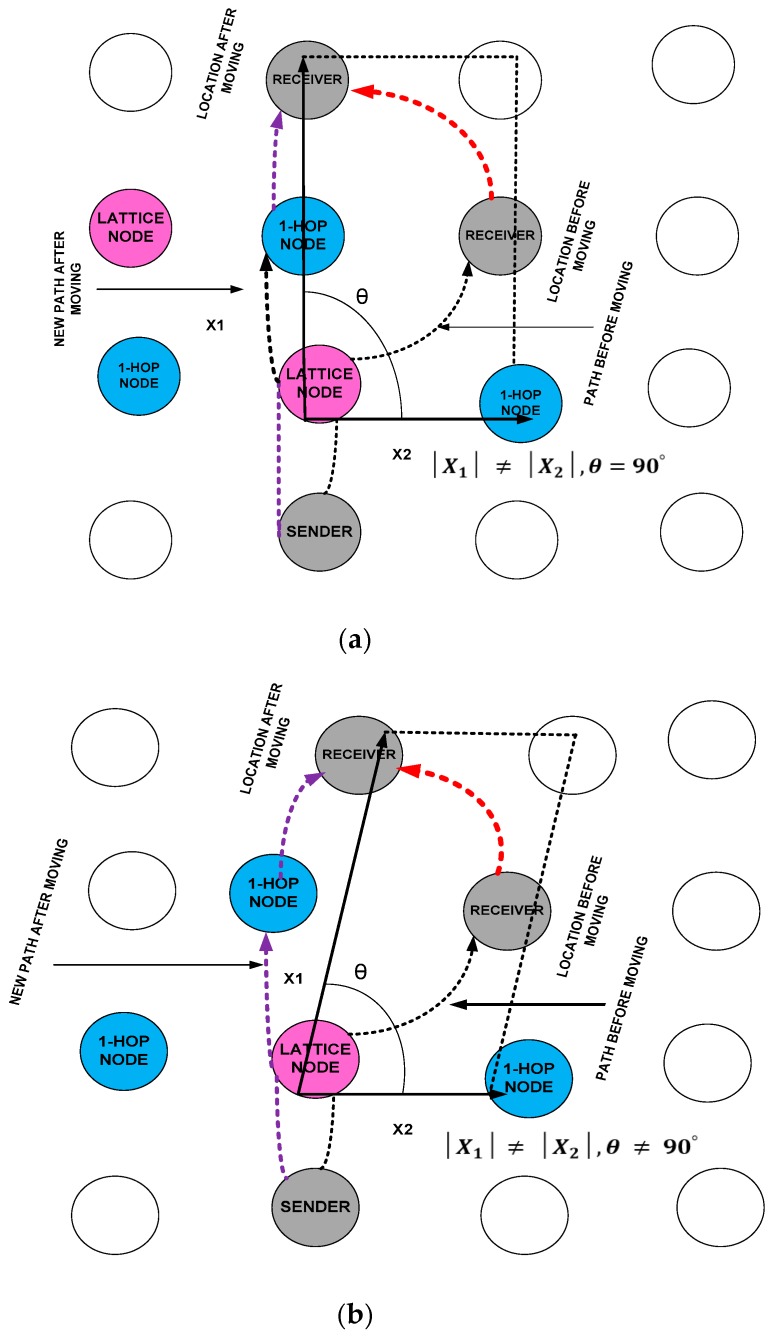

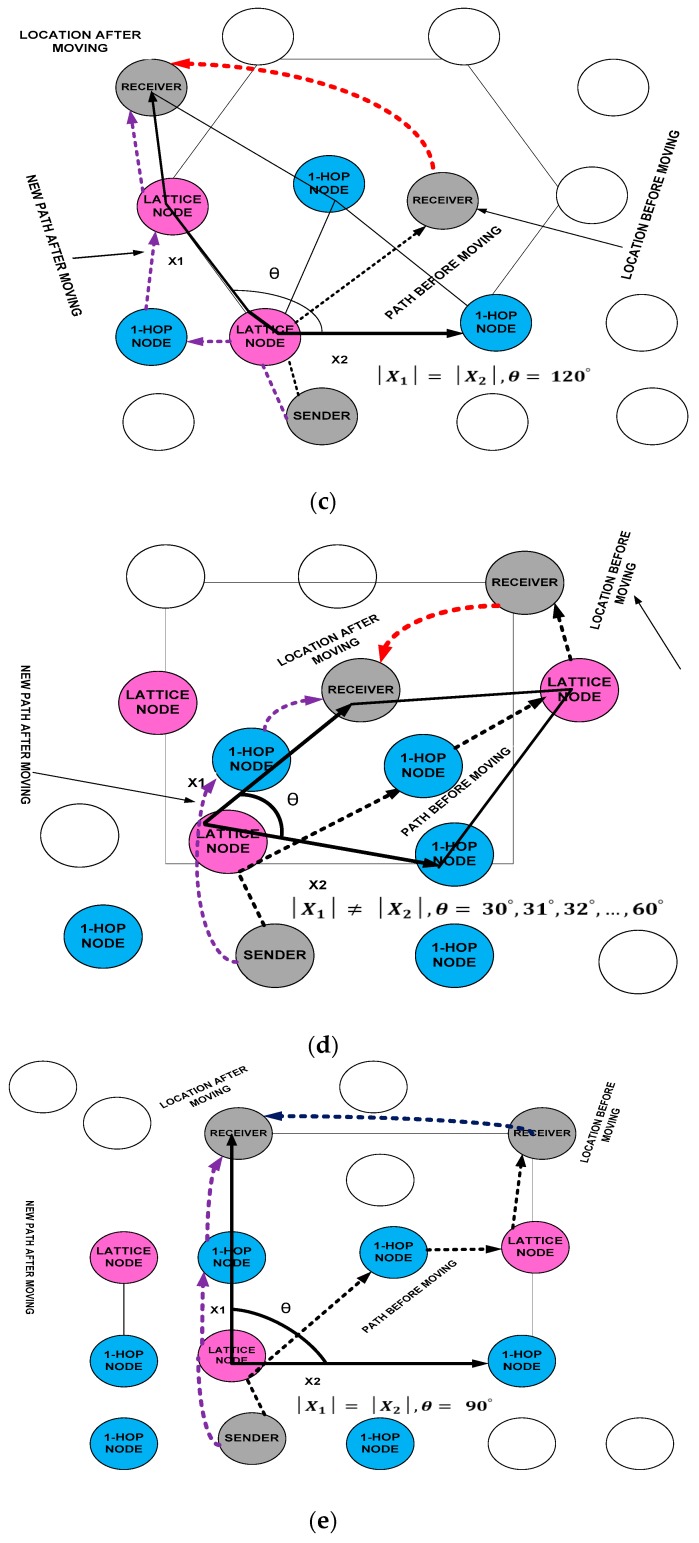
(**a**) Discovery of the new location of a moving sensor node using the rectangular Bravais lattice. (**b**) Discovery of the new location of a moving sensor node using the obliqueBravais lattice. (**c**) Discovery of new location of moving sensor node using hexagonalBravais lattice. (**d**) Discovery of the new location of a moving sensor node using the rhombicBravais lattice. (**e**) Discovery of the new location of a moving sensor node using the squareBravais lattice.

We measure the walking patterns in two dimensions, which involve five types of Bravais lattices: rectangular, oblique, hexagonal, rhombic, and square. These five Bravais lattices help determine the location of a sensor node. We apply the Fourier transform to Equation (10) to determine the accurate location of the moving sensor node. In the case of the rectangular Bravais lattice, the new location of the mobile sensor node is depicted in Figure 3a and obtained by multiplying both sides by exp(2πiC×L/M) and adding over *L*:

(12) $$\psi \left(L,\;T_{n}\right)-T_{n}\nabla \left(C\right)\psi \left(C,T_{n}\right)=1$$

where C: Lattice points on the cell corners; M: The number of dimensions

(13) $$\mathrm{with}\;\psi \left(L,\;T_{n}\right)=\sum_{L}\exp[2\pi iC\times L]\psi \left(L,T_{n}\right)$$

In the rectangular lattice, the node moves to the new location and creates a $90^{{}^{\circ}}$ angle from the distance of lattice node, but does not meet the *x*-axis and *y*-axis coordinate points.

The oblique is another type of Bravais lattice that helps determine the location of mobile sensor node when it creates a trajectory smaller than $90^{{}^{\circ}}$. Accordingly, the new location of the moving sensor node is obtained slightly differently.

Its curve is depicted in Figure 3b and obtained by:

(14) $$\psi \left(L,\;T_{n}\right)=\underset{L}{\sum }T\left(L\right)\exp[(\frac{2\pi iC\times L)}{M}]$$

The hexagonal Bravais lattice is used to determine the location of a mobile sensor node when the trajectory at the new location is exactly $120^{{}^{\circ}}$. Thus, the new location of a moving sensor node is obtained by applying the Fourier inverse of $\psi \left(C,\;T_{n}\right)$. The location of a mobile sensor node is depicted in Figure 3c.

(15) $$\psi \left(L,\;T_{n}\right)=\frac{1}{L^{2}}\underset{C}{\sum }\frac{\exp\left(-2\pi iC\times \frac{L}{M}\right)}{1-T_{n}\nabla \left(\theta \right)}$$

The rhombic is the type of Bravais lattice that can be used to find the location of a mobile sensor node when its trajectory is between $30^{{}^{\circ}}$ and $60^{{}^{\circ}}$. Hence, the new location of mobile sensor node is obtained by using the following integrands, and the trajectory of the mobile sensor node is depicted in Figure 3d:

(16) $$\psi \left(L,T_{n}\right)=\frac{1}{{\left(2\pi \right)}^{2}}\overset{-\pi }{\underset{\pi }{∭}}\frac{\exp\left(-i\theta \times L\right)}{1-T_{n}\nabla \left(\theta \right)}\;d\theta$$

The last type of two dimensional Bravais lattices is the square, which can be used to locate a mobile sensor node when its trajectory is larger than $90^{{}^{\circ}}$ and it satisfies the *x*-axis and *y*-axis coordinate points. Therefore, the trajectory of the mobile sensor node is depicted in Figure 3e and its location is obtained as:

(17) $$\psi \left(L,T_{n}\right)=\frac{1}{{\left(2\pi \right)}^{2}}\overset{-\pi }{\underset{\pi }{∭}}{\left[\nabla (\theta )\right]}^{K}\exp\left(-i\theta \times L\right)d\theta$$

The transition probability ‘*T*(*L*)’ is used to determine the distance from the original location of the moving node to its current location using the lattice structure. A simple cubic lattice has the following property:

(18) $$T\left(L\right)=\{\begin{array}{c} \frac{1}{4}\;for\;L=\left(0,\;0\;\pm 1\right),\;\left(0,\;\pm 1,\;0\right),\;\left(\pm 1,0,0\right)\\ 0\;otherwise \end{array}$$

Therefore, we can determine the exact current distance to the sensor node as:

(19) $$\nabla \left(\theta \right)=\frac{1}{4}\left(\tau^{I\theta 1}+\tau^{-I\theta 1}+\tau^{I\theta 2}+\tau^{-I\theta 2}\right)$$

(20) $$\frac{1}{4}\left(\tau^{I\theta 1}+\tau^{-I\theta 1}+\tau^{I\theta 2}+\tau^{-I\theta 2}\right)=\frac{1}{2}\left(cos\theta_{1}+cos\theta_{2}\right)$$

Thus:

(21) $$P_{K}\left(L\right)=\frac{1}{{\left(2\pi \right)}^{2}}\overset{\pi }{\underset{-\pi }{∭}}{\left[\frac{1}{2}\left(cos\theta_{1}+cos\theta_{2}\right)\right]}^{K}\exp\left(-i\theta +L\right)d\theta$$

(22) $$\mathrm{and}\;\psi \left(L,\;T_{n}\right)=\frac{1}{{\left(2\pi \right)}^{2}}{∭}_{\pi }^{-\pi }\frac{\exp\left(-i\theta \times L\right)}{1-\frac{1}{2}T_{n}\left(cos\theta_{1}+cos\theta_{2}\right)}d\theta$$

For *L* = 0, we set:

(23) $$\psi \left(L,\;T_{n}\right)=\frac{1}{{\left(2\pi \right)}^{2}}\overset{-\pi }{\underset{\pi }{∭}}\frac{d\theta_{1}}{1-\frac{1}{2}T_{n}\left(cos\theta_{1}+cos\theta_{2}\right)}\;d\theta$$

Equation (23) provides the actual distance between the original location of the moving sensor node and its current location. Let us now find the distance of the moving node from its current location to the final destination. We assume that the final distance is ∞, so L→∞. Therefore:

(24) $$\psi \left(L,\;T_{n}\right)\sim \;\frac{1}{{\left(2\pi \right)}^{2}}\overset{-\pi }{\underset{\pi }{∭}}\frac{\exp\left(-i\theta \times L\right)d\theta }{1-T_{n}+\frac{1}{4}T_{n}\;\left(cos^{2}\theta_{1}+cos^{2}\theta_{2}\right)}$$

(25) $$\begin{array}{c} \psi \left(L,\;T_{n}\right)\sim \;\frac{1}{{\left(2\pi \right)}^{2}}\overset{-\pi }{\underset{\pi }{∭}}\frac{\exp\left(-i\theta \times L\right)d\theta }{1-T_{n}+\frac{1}{4}T_{n}\;\left(cos^{2}\theta_{1}+cos^{2}\theta_{2}\right)}\\ \;=\frac{2}{2\pi T_{n}L}\exp\{-2^{\frac{1}{2}}L{\left[\frac{2\left(1-T_{n}\right)}{T_{n}}\right]}^{\frac{1}{2}}\} \end{array}$$

where L=|L|.

The detail of used notations and descriptions is given in Table 1.

Thus, the total energy ‘E’ consumed in updating the locations $L_{u}$ of the sink nodes and the cost for each complete cycle (Even-monitoring process) $C_{c}$ used in [23] can be given by:

(26) $$L_{ue}=\overset{N}{\underset{i=1}{\sum }}e(K_{n})\times C_{m}$$

Equation (26) denotes the energy consumed for the location update:

(27) $$E_{s}=\frac{n\times \left(∆C_{p}\times R_{e}\right)+n\times \left(A_{e}\times C_{m}\right)}{2M_{e}}+r^{2}\;\left(K_{n}-1\right)$$

Equation (27) shows the consumed energy for synchronization:

(28) $$E_{d}=\frac{{\left\{n\times \left(∆D_{p}\times R_{e}\right)+n\times \left(A_{e}\times C_{m}\right)\right\}}^{2}}{2M_{e}}+r^{2}\;\left(K_{n}-1\right)$$

Equation (28) shows the consumed energy for receiving and forwarding the data to the next node:

(29) $$\begin{array}{c} C_{c}=\left\{\frac{{\left\{n\times \left(∆D_{p}\times R_{e}\right)+n\times \left(A_{e}\times C_{m}\right)\right\}}^{2}}{2M_{e}}+r^{2}\;\left(K_{n}-1\right)\right\}\\ \;+\left\{\frac{n\times \left(∆C_{p}\times R_{e}\right)+n\times \left(A_{e}\times C_{m}\right)}{2M_{e}}+r^{2}\;\left(K_{n}-1\right)\right\} \end{array}$$

By combing the Equations (26) and (29), we get the total energy consumed for updating the location and each complete cycle as given by:

(30) $$E=L_{ue}+C_{c}$$

This model helps accurately determine the moving location, the distance from the original location of the sensor node to the current location. Additionally, it reduces control packets by applying the optimized data frame format model approach discussed in [23], energy consumption and end-to-end delay. The mobile sensor nodes in LMM sense the event from the target location and transfer the generated events to the base station. If any sensor node moves from its original position, another node fills the gap. The location of the moving node is determined to manage the data transmission rate.

The lattice provides scalability to WSNs because the locations and distances of the moving sensor nodes are easily determined to control the mobility. The LMM is suitable for single-hop destinations, but its performance on multi-hop destinations has not been tested. Moreover, multiple hops on the path create nonlinearities in the WSN. Before a packet can be transmitted, a node should wait for the node of the next hop to wake up. The packet is held for a variable amount of time on every path. This feature creates uncertainty for several types of WSN applications, especially surveillance and military applications.

The LMM is a memory less model because WSNs have sensor nodes with minimum memory and power. The lower energy consumption for mobility helps gather additional data due to a longer network life. We focus on other lower-memory mobility models, including the geographic based CMM, WMM, and RWMM. The performances of these mobility models are compared in the realistic scenario of an earthquake, which is simulated in the next section.

## 4. Simulation Setup and Analysis of Results

We simulate LMM using OMNet++ [24]. The network contains 500 sensor nodes that are randomly deployed over a 1600 × 1600 square meter field. Initially, the border node remains in a corner of each region and movable sensor nodes are placed at the corners of the regions. When the simulation begins, the mobile sensor nodes move back and forth in the network regions. Each simulation run lasts for 14 min, and three mobility models are tested: CMM, WMM, and RWMM. We use the pheromone termite routing protocol to handle the packet generation rate and pheromone sensitivity used in [25].

At the transport layer, packet re-ordering is a major issue, particularly for multimedia contents [26], so we use Reliable Multi Segment Transport protocol (RMST) [27] to help re-order the packet, which guarantees consistent pre-packet delivery for different packet types and enables faster recovery in case of packet loss. The packet size is set to 128 bytes for file transfer and 512 bytes for video, audio, and image transfer. We also consider a sensor application module with a constant bit-rate source that maintains QoS requirements. The contending algorithms CMM, WMM, and RWMM have comparable accuracy and tune our proposed LMM algorithm to have the similar accuracy in order to compare the performance. The detailed simulation parameters are presented in Table 2.

We obtained several results, but used the following metrics to demonstrate the performance of the LMM in the WSN:

▪ Average delay and average power consumption
▪ The times taken by the sensor nodes to reach different positions using the LMM, CMM, RWMM, and WMM at fixed and variable rates of motion.
▪ The drop rates using the LMM, CMM, RWMM, and WMM at different rates of motion.
▪ The residual energy of the nodes and border node after changing the locations using the LMM, CMM, RWMM, and WMM.

**(A) Average delay and average power consumption**

**Figure 4:**
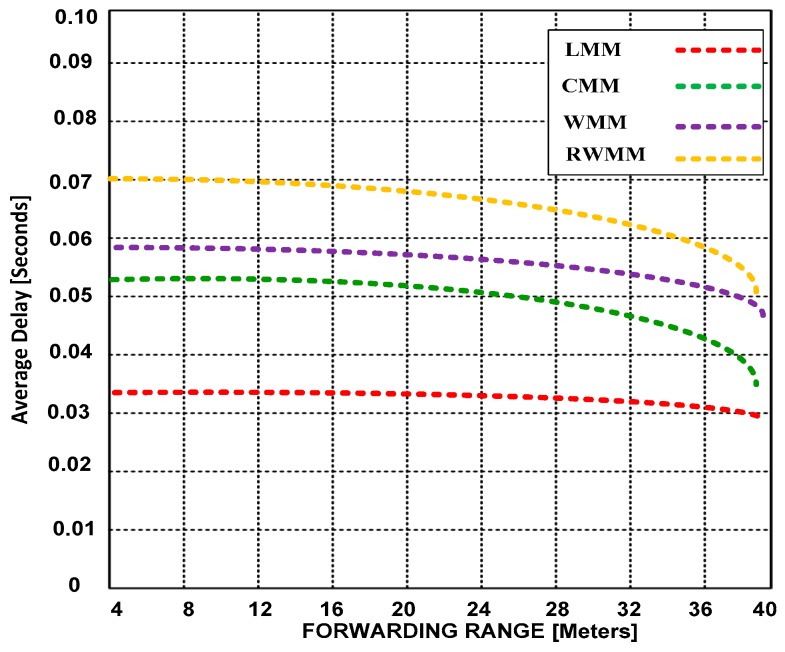
Average delay versus forwarding range at 25 m event range and 50% mobility.

**Figure 5:**
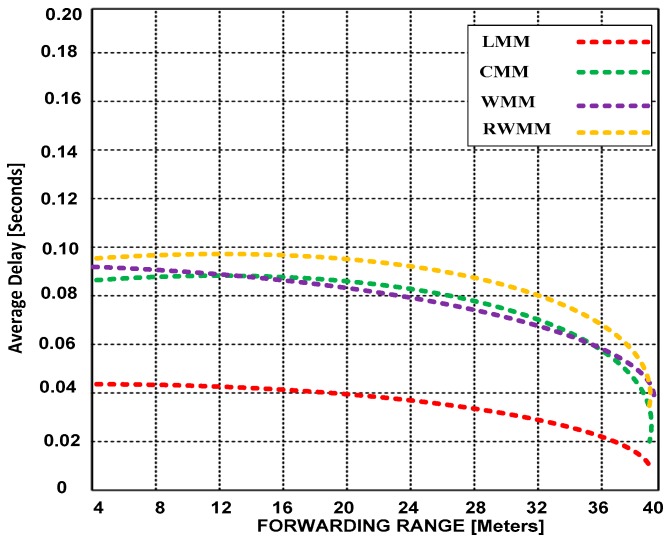
Average delay versus forwarding range at 50 m event range and 50% mobility.

In Figure 4 and Figure 5, we show the average power consumption based on increased event range and forwarding range. The sensor node within an event area reports to the border node in each region. The event area is centered at (300, 300) meters. The figures show the simulation runs for the high moderate traffic rate. The mobility is set to 50% and an event range is 25 and 50 m around the center in Figure 4 and Figure 5, respectively. There are 200 sensor nodes, which participate in the event and use 42 sources (activities performed in each event).

In Figure 4 and Figure 5, we demonstrate that in the situation of high moderate traffic rate, which is now common in WSNs due to the emergence of new mobility based applications, reducing end-to-end delay is of paramount significance for obtaining improved throughput. Thus, we validate that end-to-end delay can be reduced by extending the forwarding range. This is a significant trade-off that has not been investigated until now. In addition, we have validated that LMM outperforms CMM, WMM and RWMM in walking mobility patterns. Furthermore, RWMM performs poorly in reduced and extended forwarding range.

**Figure 6:**
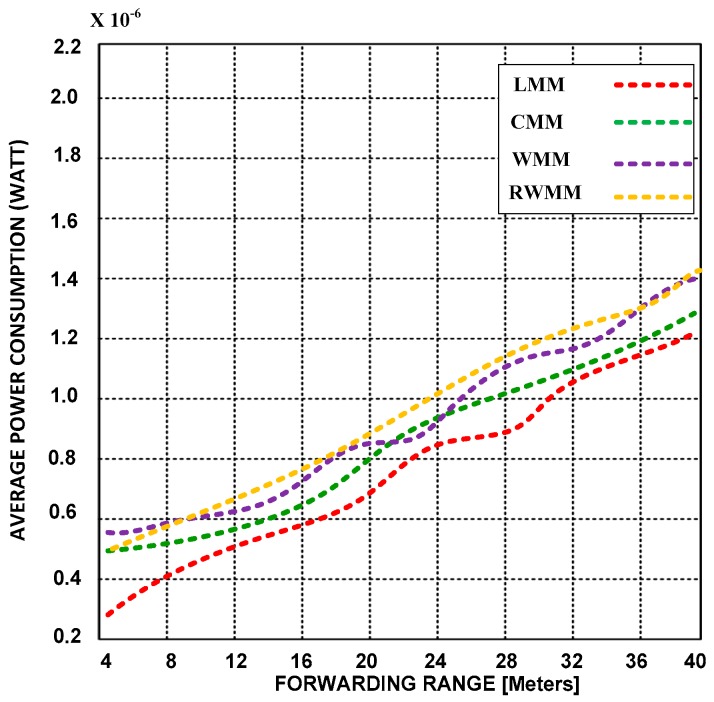
Power consumption versus forwarding range at 25 m event range and 50% mobility rates.

**Figure 7:**
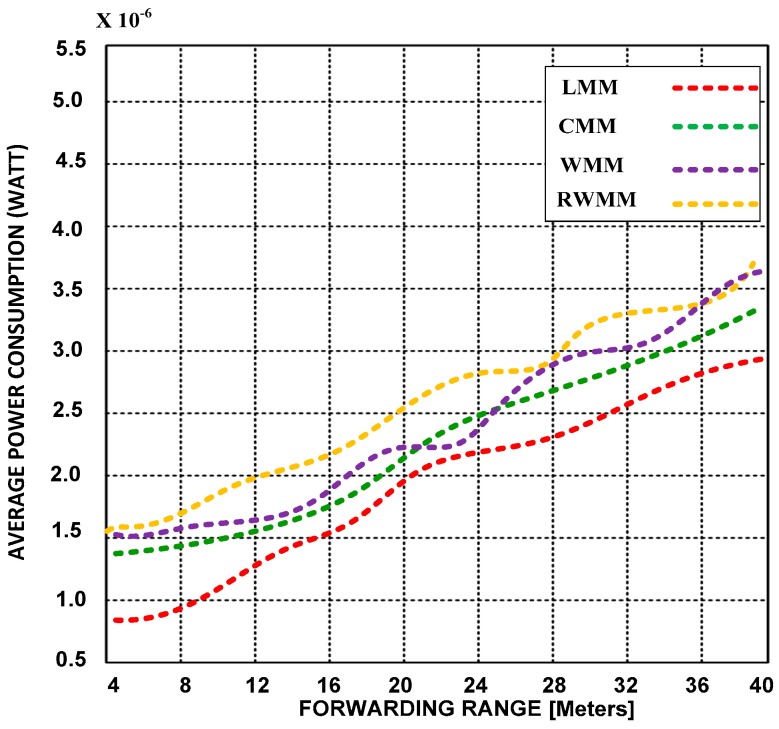
Power consumption versus forwarding range at 50 m event range and 50% mobility rates.

In Figure 6 and Figure 7, we show an average power consumption using short and long event distances and forwarding ranges. 

The event area, the rate of traffic load, event ranges, forwarding ranges, number of participant nodes inside the event, traffic sources, and mobility parameters are the same as those mentioned in Figure 4 and Figure 5.

The simulation results demonstrate that extended forwarding range does not affect power consumption or latency. However, LMM consumes less power in short and extended forwarding ranges than CMM, RWMM and WMM. The RWMM also consumes more power than the other participants’ mobility models. We also validated based on simulation results that RWMM is not best choice for WSNs from a latency and power consumption point of view.

**(B) Time taken by sensor nodes to reach different locations**

**Figure 8:**
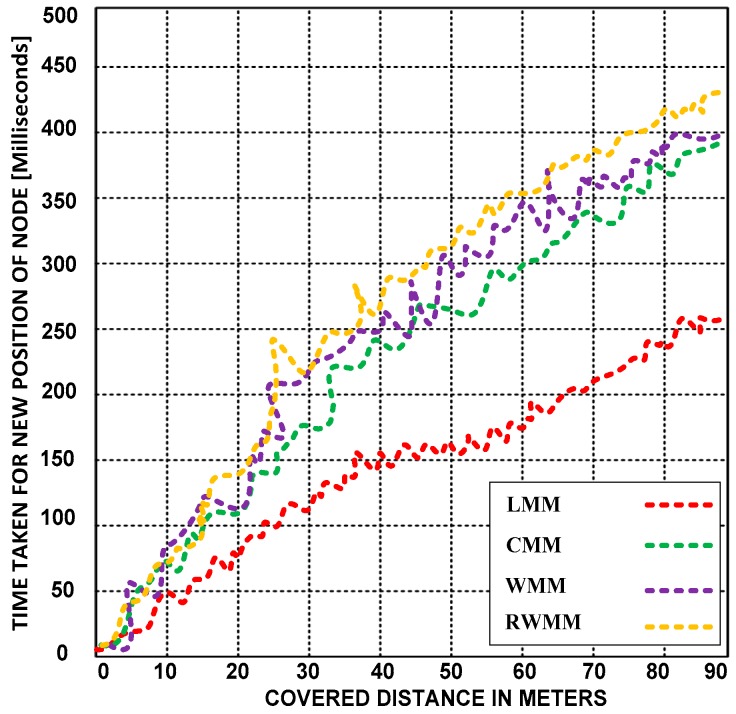
Times taken by the sensor node to reach different positions using the LMM, CMM, RWMM, and WMM at a fixed velocity of 10 m/s.

Here, we examine the compatibility of the LMM with sensor nodes. Based on the simulation results, we analyze the time taken for each moving node to reach different positions. We also compare the efficiency of the LMM with the CMM, WMM, and RWMM. Figure 8 presents the distances covered by the sensor nodes using the LMM and the other mobility models. 

The LMM is more compatible with sensor nodes because it takes less time to change location than the other mobility models. Before letting the sensor nodes move to other locations, the LMM collects several pieces of information, including the moving location, the distance from the original location to the current location, and the distance from the current location to the destination.

**Figure 9:**
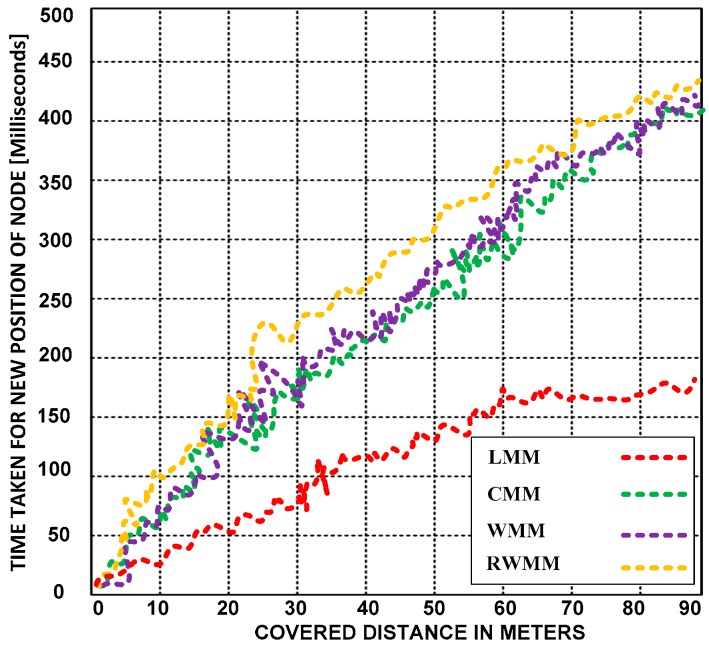
Times taken by the sensor node to reach different positions using the LMM, CMM, RWMM, and WMM at different velocities.

Figure 9 presents the moving times of the sensor nodes using the LMM and the other mobility models at different velocities. The LMM outperforms the other mobility models; it is scalable because the locations and the distance between the moving sensor nodes are easily determined, and the motion only marginally increases the time required to change positions.

**(C) Drop Rate using the LMM, CMM, RWMM, and WMM**

Network congestion and a lack of network coverage are key factors that affect the drop rate of packets. These two issues are directly or indirectly dependent on the performance of the mobility models. Congestion and lack of coverage prevent packets from successfully arriving at the base station. The coverage problem includes monitoring a set of goals in the intended area. The sensor nodes collect the information from events within their communication range, and forward it to the base station. The base station may not be able to receive transmitted packets due to such coverage problems. 

**Figure 10:**
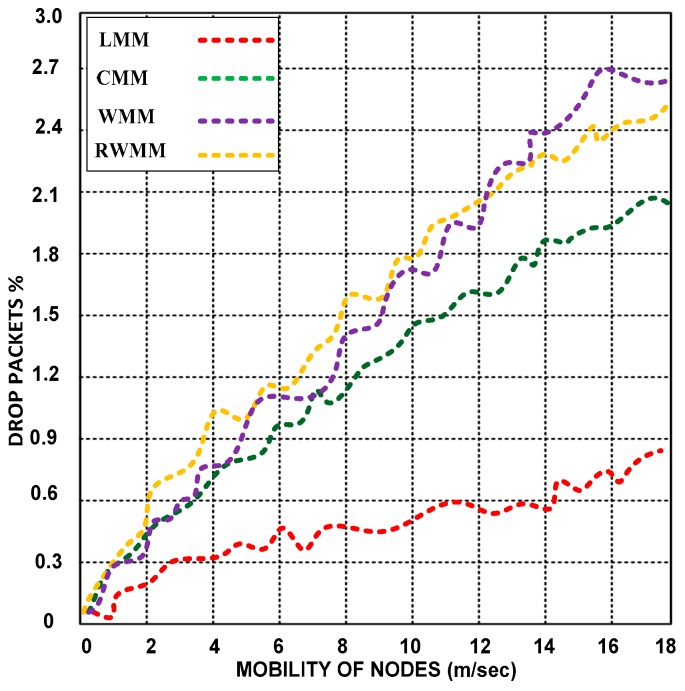
Packet drop rates using the LMM, CMM, RWMM, and WMM at different velocities.

Figure 10 compares the packet drop rate of the LMM with those of the CMM, RWMM, and WMM. The LMM outperforms the other mobility models in walking patterns; it drops a maximum of 0.7% of the packets, whereas the other mobility models have dropped rates as high as 2.45%. This result confirms the high potential of the LMM as a mobility model for walking patterns.

**(D) Residual energy**

**Figure 11:**
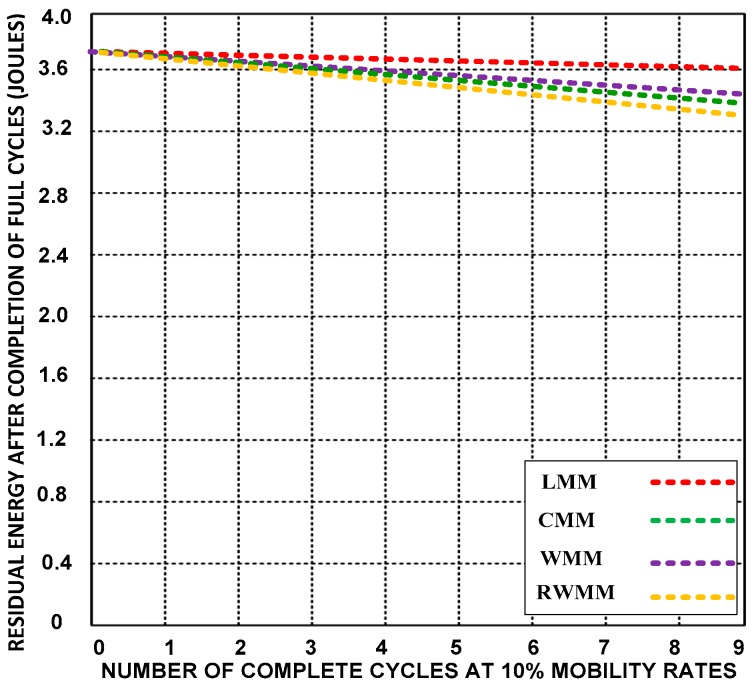
Residual energy after completion of the ninth cycle at 10% mobility rates.

**Figure 12:**
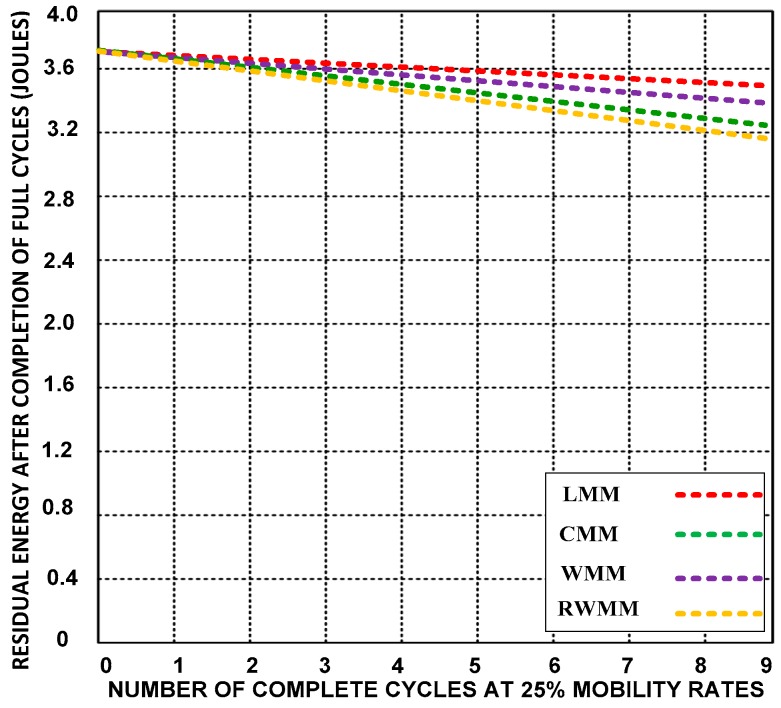
Residual energy after completion of the ninth cycle at 25% mobility rates.

**Figure 13:**
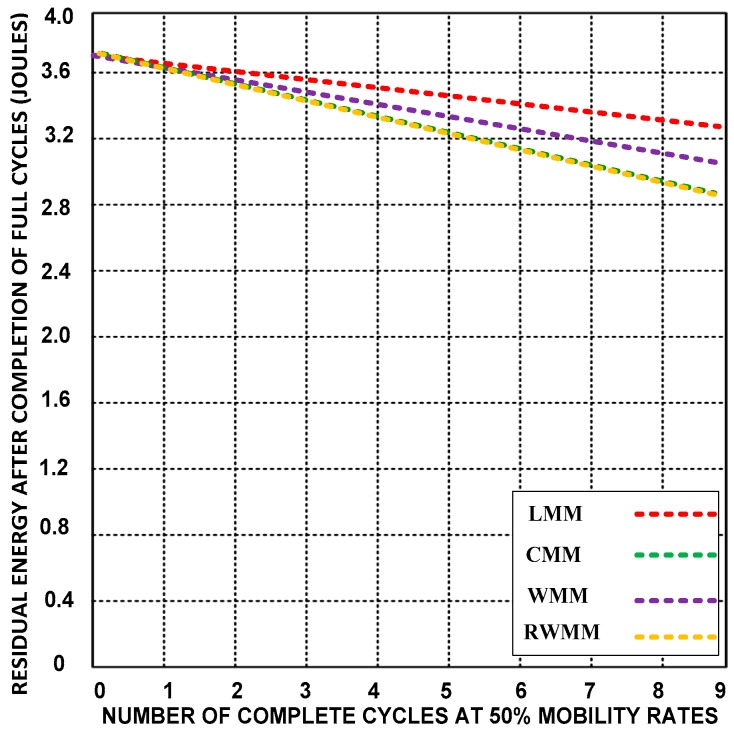
Residual energy after completion of nine cycles at 50% mobility rates.

Residual energy is the remaining energy level of the sensor nodes after completing the task. Here, we discuss the average residual energy level of the sensor nodes after changing locations. Figure 11, Figure 12 and Figure 13 compare the residual energy of LMM with those of the CMM, RWMM, and WMM at 10%, 25% and 50% mobile sensor nodes. The sensor nodes have a higher residual energy with the LMM after changing location seven times.

The residual energy of the LMM decreases from 0.12 to 0.38 Joules at 10%, 25% and 50% mobility rates, which is a smaller decrease equivalent to 10.27% after completion of nine cycles than for the other mobility models. The energy levels of the other mobility models decrease from 0.28 to 0.756 Joules after completing ten motion cycles (the completion of event monitoring), which is equivalent to 20.27%. It is almost a double energy consumption waste.

**(E) Time Complexity**

The better performance of mobility model depends on the minimum time complexity. The time complexity is measured by manipulating the number of basic operations performed by the mobility model and constant amount of time to accomplish. Our proposed LMM gives lower time complexity as compared to other mobility models. LMM and competing mobility models are based on recursive approach. Therefore, time complexity of mobility models can be obtained using recursive approach shown in Table 3.

(31) $$T\left(n\right)=\{\begin{array}{c} O\left(1\right)\;If\;n=1\\ at\left(\frac{n}{b}\right)+O\left(n\right)\;If\;n>1 \end{array}$$

## 5. Discussion of Results

The state-of-art research on WSNs depends on energy consumption for maintaining QoS provisioning. The energy consumption due to effect of the mobility model has been significant trade-off that has not fully been explored. The LMM produces better residual energy because of the memory less model, which supports the sensor nodes due to their low battery power. Furthermore, LMM has a marginal chance of breaking the links and MAC failure.

Based on the results, we demonstrate the performance of LMM and show in Table 4 its comparison with other mobility models: CMM, WMM and RWMM. The overall results validate that LMM has minimum average delay 0.030 and 0.008 at 25 and 50 event ranges that is much lower than CMM, WMM and RWMM models. LMM consumes less power (1.21 × 10^−6^ and 2.28 × 10^−6^ at 25 and 50 m event ranges) as compared with other contending mobility models. Once nodes change the locations using LMM at 10 m/s and variable velocities, then minimum time is required as compared to other competing mobility models. LMM produces lower drop rate that is 0.78% from 0 to 18 m/s velocities. The mobility affects the performance of the network because nodes start dropping the energy, as the result, throughput is reduced. The LMM is not highly affected that is reason, it possesses the better residual energy 3.7, 3.51 and 3.32 joules with 10%, 25% and 50% mobility rates respectively as compared to other contending mobility models. The LMM overcomes the shortcomings of competing mobility models by providing the better results in the state-of-the-art performance-metrics. As, LMM deals with the node and sink mobility synchronously. Furthermore, it reduces the control packets and pause time to obtain higher throughput performance. The LMM is suitable for those applications where events are required mobility-supported sensor nodes to accomplish the tasks specially disaster-recovery, battlefield, cool-mining monitoring, animal movement detection etc., Based on the overall result, LMM has edge over other competing mobility models. Table 5 demonstrates the improvement of LMM over CMM, WMM and RWMM mobility models.

## 6. Conclusions

This paper presents a mobility model, LMM, for walking patterns over WSNs. To validate the strength of LMM, the realistic scenario of an earthquake disaster recovery is simulated using OMNet++. The LMM determines the motion of the nodes and accurately provides the node’s moving location, the distance from the original location of the sensor node to its current location, and the distance from its current location to its destination. We simulated the mobility scenarios and measured the performance in terms of QoS provisioning. Based on the simulation results, we discovered that latency, one of the most important QoS parameters, can be reduced by increasing the forwarding range. This is a significant trade-off that has not yet been investigated. The statistical data obtained through simulation is also evidence of the strength of LMM, as it outperformed the CMM, WMM, and RWMM in end-to-end delay, time taken by nodes to reach their next locations, drop rate, and residual energy at variable mobility rates. The LMM decreases the time for the sensor node to change its location by 34.2–52.8%. In addition, the LMM has a lower drop rate, 0.7% than other mobility models, which have drop rates as high as 2.45%. LMM decreases energy from 0.12 to 0.38 joules at 10%, 25% and 50% mobility rates—that is 10.27% energy consumption at ten complete motion cycles. However, CMM, WMM, and RWMM decrease energy from 0.28 to 0.756 Joules—that is 20.27%. These results demonstrate that LMM is an ideal choice for different kinds of walking patterns over WSNs. In the future, we will simulate dynamic medium mobility patterns, and vehicular mobility patterns using LMM to determine its suitability.

## Figures and Tables

**Table 1:** Notations and its descriptions.

Notations	Description
*a*	Neighbor node
$a_{i}$	Number of neighbor nodes
$A_{e}$	Energy consumed for amplifying the signal
$C_{m}$	Control message for location update
*C*	Lattice points on the cell corners
*D*	Node distribution capability
$D_{i}$	Node distribution probability for entire network
∆*di*	Different directions of the neighbor nodes
$D_{I}{}^{\beta }$	the distribution of the nodes that can be predicted by the value of $D_{i}\left(a,t\right)$
e	Energy consumed for updating the location to single node
*E*	Total Energy consumed for updating the location and cycle
$E_{d}$	Energy consumed for sending and receiving the data
$E_{s}$	Energy consumed for synchronization
$∆C_{p}$	Control packets
*K*	Sensor node
$K_{n}$	Number of sensor nodes
*L*	Location
$L_{ue}$	Energy consumed for location update
*M*	The number of dimensions
*n*	The number of messages
$2M_{e}$	Mean Energy consumed for radio and amplifying the signal
*PK* (*L*)	Probability of determining the location of a sensor node
*R*	Region
$R_{e}$	Energy consumed for the radio signal
γ	Upper bond
T(L−Ĺ)	Probability of the sensor node when it moves from location ‘*L*’ to location ‘Ĺ’
Rinfmum	The sensor node possesses infimum feature
RSupmum	The sensor node possesses supremum feature
Rhigh	Highest residual energy of node in the region
Rlow	Shortest distance of the node from sender in the region
*t*	time
$T_{n}$	Trajectory of a sensor node
*V*	Speed of mobile sensor node
$X=(X_{1},\;X_{2})$	Locations
$\tau_{i}$	Different speeds of each mobile sensor node
ψ	state of the node in WSNs
*i*	Indicates the number of the mobile sensor nodes having an average speed

**Table 2:** Simulation parameters and corresponding values.

Parameters	Value
Size of WSN	1600 × 1600 m^2^
Size of each region	400 × 400 m^2^
Number of nodes	500
Routing Protocols	Pheromone termite routing protocol
Medium Access Control Protocol	BN-MAC
Transport layer Protocol	Reliable Multi Segment Transport protocol (RMST)
Queue-Capacity	30 Packets
Mobility Model	WMM, RWMM, CMM, and LMM
Maximum number of retransmissions allowed	03
Event distances	25 and 50 m
Size of Packets	128 bytes for file transfer and 512 bytes for video, audio, and images
Data Rate	300 kilobytes/s
Time for topology change	1.2 s
Propagation model	Deterministic
Sensing Range of node	15 m
Forwarding range	4–40 m
Maximum bandwidth of node	250 kilobytes/s
Simulation time	14 min
Average Simulation Run	15

**Table 3:** Time complexity for SDAAA and contending algorithms.

Mobility Models	Time Complexity Derivations
**LMM**	$T\left(n\right)=at\left(\frac{n}{b}\right)+O\left(n\right)$ Model uses finite set of inputs and time complexity remains constant ‘*n*’ $T\left(n\right)=t\left(\frac{n}{2}\right)+O\left(n\right)$ $T\left(n\right)=t\left(\frac{n}{2}\right)+n$ $\left(n\right)=t\left(\frac{n}{n}\right)+n$ T(n)=t(1)+n T(n)=t+n Ignore ‘*t*’ T(n)=n As, we know that *n* = *k* & *k* = *log n* Thus, time complexity is O(log n)
**CMM**	$T\left(n\right)=at\left(\frac{n}{b}\right)+O\left(n\right)$ Model uses finite set of inputs and time complexity linearly increases. As, we get: $T\left(n\right)=t\left(\frac{n}{2}\right)+O\left(n\right)$ $T\left(n\right)=t\left(\frac{n}{n}\right)+O\left(n\right)$ T(n)=t+O(n) Where ignore ‘t’; Thus, T(n)=O(n)
**WMM**	$T\left(n\right)=at\left(\frac{n}{b}\right)+O\left(n\right)$ Model uses finite set of inputs and time complexity remains constant ‘*n*’ $T\left(n\right)=t\left(\frac{n}{2}\right)+O\left(n\right)$ $T\left(n\right)=t\left(\frac{n}{2}\right)+n+n$ $\begin{array}{c} .\\ . \end{array}$ $\begin{array}{c} .\\ . \end{array}$ $\left(n\right)=t\left(\frac{n}{n}\right)+n+n$ T(n)=t(1)+n+n T(n)=t+n+n Ignore ‘*t*’ T(n)=n+n *N* = *k* & *k* = *log n* substitute single ‘*n*’ then we get: *O*(*log n* + *n*)
**RWMM**	$T\left(n\right)=at\left(\frac{n}{b}\right)+O\left(n\right)$ The model divides the task into parts with different size $T\left(n\right)=t\left(\frac{n}{3}\right)+t\left(\frac{2n}{3}\right)+O\left(n\right)$ $T\left(n\right)=t\left(\frac{n}{3}\right)+t\left(\frac{2n}{3}\right)+O\left(n\right)$ $T\left(n\right)=t\left(\frac{n}{3}\right)+t\left(\frac{2n}{3}\right)+n+n$ T(n)=2t+2n T(n)=lognn T(n)=O(nlog(n))

**Table 4:** Compression of LMM with other competing mobility models: CMM, WMM and RWMM.

Mobility Models	Average Delay		Average Power Consumption in Watt		Change Location Time		Drop Rate	Residual Energy		
	Event Range 25 m	Event Range 50 m	Event Range 25 m	Event Range 50 m	With fixed 10 m/s Velocity	Variable Velocities	0–18 m/s Velocities	10% Mobility	25% Mobility	50% Mobility
**LMM**	0.030 s	0.008 s	1.21 × 10^−6^	2.28 × 10^−6^	252 Milliseconds	177 Milliseconds	0.78%	3.7 Joules	3.51 Joules	3.32 Joules
**CMM**	0.038 s	0.020 s	1.29 × 10^−6^	3.33 × 10^−6^	383 Milliseconds	409 Milliseconds	1.99%	3.42 Joules	3.34 Joules	2.84 Joules
**WMM**	0.048 s	0.039 s	1.39 × 10^−6^	3.62 × 10^−6^	398 Milliseconds	417 Milliseconds	2.64%	3.44 Joules	3.41 Joules	3.02 Joules
**RWMM**	0.051 s	0.038 s	1.41 × 10^−6^	3.64 × 10^−6^	464 Milliseconds	428 Milliseconds	2.51%	3.31 Joules	3.16 Joules	2.84 Joules

**Table 5:** LMM-improvement over other competing mobility models.

Parameters	Improvement in LMM as Compared with CMM, WMM and RWMM
Drop Rate	1.21–1.73%
Average Power Consumption (Watt)	14.285%
Node Location finding capability	34.2%–52.8%
Energy Consumption with different mobility rates	3.2–10%
Average Delay	8–21%

## Appendix A

**Definition** **A1.**
*A region ‘R’ is the set of sensor nodes; thus, it can be expressed as R ⊆ N of sensor nodes in network ‘N’ is bounded from above if there exists a sensor node ‘K’ in region ‘R’ such that K ∈ N is an upper bound of ‘R’. K ∈ N is an upper bound of ‘R’ if there exists a sensor node ‘K’ in region ‘R’ such that for the remaining sensor nodes ‘γ’ in region ‘R’, γ ≤ K for every γ ∈ R. This helps determine the node with the highest residual energy in the region ‘R’ at a one-hop neighborhood destination. Similarly, ‘R’ is bounded from below if there exists a K ∈ N, called the greatest lower bound of ‘R’, such that γ ≥ K for every γ ∈ R. Thus, a single node is selected from the list of three sensor nodes based on the shortest distance (using infimum) and the highest residual energy (using supremum). If both conditions are satisfied, then this search continues until it reaches the desired moving sensor node for discovery of a location and path.*

**Definition** **A2.**
*Suppose that R ⊆ N consists of sensor nodes available in the region ‘R’, which is a subset (part) of the network ‘N’. If K ∈ N is the upper bound of ‘R’ such that K ≥ Ḱ for every upper bound in region ‘R’, where ‘Ḱ’ is the list of sensor nodes with lower upper bound; then ‘K’ is called a supremum in region ‘R’, represented by K = RSupmum. If K ∈ N is the greatest lower bound in the region ‘R’ such that K ≤ Ḱ for every greatest lower bound, then ‘K’ is called the greatest lower bound or infimum (a node with the shortest distance from the transmitting node) in the region ‘R’, represented by K = Rinfmum.*

*If ‘R’ is not bounded, then we can write RSupmum = −∞. If R = { } is the empty region, then every sensor node is a greatest lower and upper bound in region ‘R’, and we can write φsupmum = −∞, φInfmum = ∞. We can conclude that the region ‘R’ has a supremum and infimum if each region consists of a finite number of nodes. For region R = {γK: K ∈ N}, it can be written as follows:*

(A1) $$R_{supmum}=\sum_{K\epsilon N}\gamma_{K}\&R_{infmum}=\sum_{K\epsilon N}\gamma_{K}$$

**Corollary** **A1.**
*The region ‘R’ consisting of the set of sensor nodes is bounded.*

**Remark** **A1.**
*If a sensor node ‘K’ is infimum (with shortest distance) at one hop neighborhood in the region ‘R’ then a sensor node ‘K’ will be the greatest lower bound in the list of remaining sensor nodes ‘γ’; thus, K ≤ γ ∈ R is the greatest lower bound.*

**Notation** **A1.**
*We can write ∆L(R) for the set of the greatest lower bounds (infimum: shortest distance at one-hop neighborhood) and ∆U(R) for set of upper bounds (supremum: Highest residual energy of one-hop neighborhood node) in the region ‘R’.*

*The above remark can be expressed as follows:*

*If K ∈ ∆L(R) and K ≤ γ, then γ ∈ ∆L(R). Similarly, if K ∈ ∆U(R) and K ≥ γ, then γ ∈ ∆U(R).*

**Definition** **A3.**
*A sensor node ‘K’ is called a supremum (A node with the highest residual energy) in the region ‘R’ at a one-hop neighborhood if*

a. *K ∈ ∆U(R)*
b. *K ≥ γ for all γ ∈ ∆U(R). i.e., ‘K’ is upper bound in ’R’.*

*Thus, a sensor node ‘K’ is called the upper bound in the region ‘R’.*

*A sensor node ‘K’ is called an infimum (A node available at the shortest distance) in the region ‘R’ if*

a. *K ∈ ∆L(R)*
b. *K ≤ γ for all γ ∈ ∆L(R). i.e., ‘K’ is the greatest lower bound in ’R’.*

*Therefore, a sensor node ‘K’ is chosen as a lattice node if both conditions are satisfied.*

**Example** **A1.**
*Let R = {K ∈ N│ 0 < K ≤ 1}. Then RSupmum = 1 &Rinfmum = 0.*

**Remark** **A2.**
*If RSupmum ∈ R, then it is also called the maximum in the region ‘R’.*

*Similarly, If Rinfmum ∈ R, then it is also called the minimum in the region ‘R’.*
